# Multi-input Synapses, but Not LTP-Strengthened Synapses, Correlate with Hippocampal Memory Storage in Aged Mice

**DOI:** 10.1016/j.cub.2019.08.064

**Published:** 2019-11-04

**Authors:** Wajeeha Aziz, Igor Kraev, Keiko Mizuno, Alastair Kirby, Ton Fang, Huzefa Rupawala, Kamillia Kasbi, Stephanie Rothe, Felix Jozsa, Kobi Rosenblum, Michael G. Stewart, K. Peter Giese

**Affiliations:** 1 Department of Basic and Clinical Neuroscience, Institute of Psychiatry, Psychology & Neuroscience, King’s College London, 5 Cutcombe Road, London SE5 9RX, UK; 2 Department of Medical Education, Brighton and Sussex Medical School, University of Brighton, Watson Building, Brighton BN1 9PH, UK; 3 Department of Life, Health & Chemical Sciences, The Open University, Walton Hall, Milton Keynes MK7 6AA, UK; 4 Sagol Department of Neurobiology, Center for Gene Manipulation in the Brain, University of Haifa, Mount Carmel, Haifa 3498838, Israel

**Keywords:** memory storage, normal aging, structural plasticity at synapses, contextual fear conditioning, reconsolidation, multiinnervated dendritic spines, synaptic signaling, CaMKII, PSD-95, nNOS

## Abstract

Long-lasting changes at synapses enable memory storage in the brain. Although aging is associated with impaired memory formation, it is not known whether the synaptic underpinnings of memory storage differ with age. Using a training schedule that results in the same behavioral memory formation in young and aged mice, we examined synapse ultrastructure and molecular signaling in the hippocampus after contextual fear conditioning. Only in young, but not old mice, contextual fear memory formation was associated with synaptic changes that characterize well-known, long-term potentiation, a strengthening of existing synapses with one input. Instead, old-age memory was correlated with generation of multi-innervated dendritic spines (MISs), which are predominantly two-input synapses formed by the attraction of an additional excitatory, presynaptic terminal onto an existing synapse. Accordingly, a blocker used to inhibit MIS generation impaired contextual fear memory only in old mice. Our results reveal how the synaptic basis of hippocampal memory storage changes with age and suggest that these distinct memory-storing mechanisms may explain impaired updating in old age.

## Highlights

• Aged mice form contextual memory like young mice, but reconsolidation is impaired
• Only in young mice is contextual memory formation associated with structural LTP
• In aged mice, contextual memory formation correlates with multi-innervated spines
• Inhibition of multi-innervated spines impairs memory in aged but not young mice

## Graphical Abstract

**Figure:**
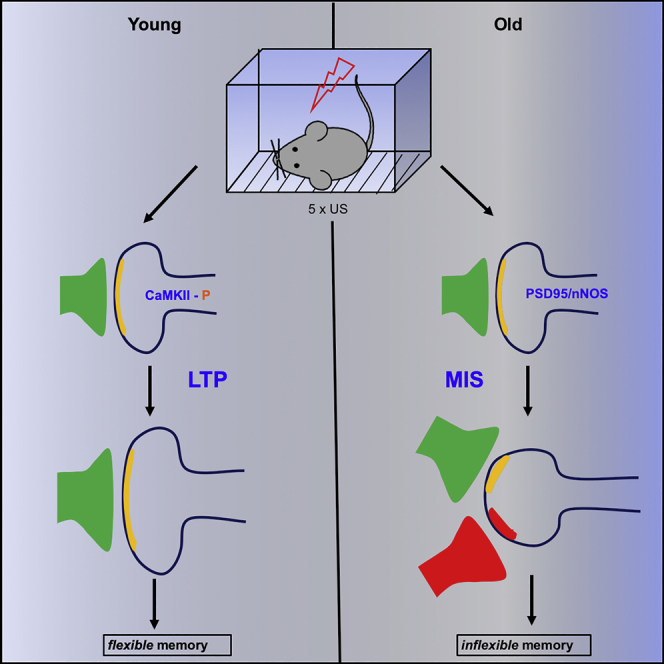

## Teaser

Aziz et al. show that the synaptic basis of contextual fear memory differs dramatically with age. In young mice, memory associates with strengthening of synapses with one synaptic input, whereas in aged mice it correlates with multi-input synapses. Memory updating in old mice is impaired, and this may be due to the distinct memory-storing mechanism.

## Introduction

Memory can be stored for a long time after going through a brief cellular consolidation period [1]. This memory storage is thought to occur at synapses, which have been strengthened through long-term potentiation (LTP) [2, 3, 4]. The synaptic changes that characterize maintenance of LTP include increased density of AMPA receptor subunits in the postsynaptic density (PSD) and enlarged synapse morphology [5, 6, 7]. Perpetuating signaling that maintains LTP is envisaged to be the molecular basis of memory storage [4, 8].

Most mechanistic studies of memory storage have focused on the hippocampus, due to its critical role in memory [9]. Hippocampal memory formation declines with aging [10, 11, 12, 13]. Despite this age-related decline, hippocampal memory can still be formed and stored, posing the question of what synaptic mechanisms enable memory storage in old age. Aging causes a deficit in LTP induction, which can be overcome with strong electrical stimulation [10, 12, 14]. Therefore, it is conceivable that extended behavioral training may overcome age-related impairment in LTP induction so that LTP may be the memory-storing mechanism, like at a young age. Alternatively, in old age, extended behavioral training might not be sufficient to induce LTP but instead may engage an alternative synaptic change, such as generation of multi-innervated dendritic spines (MISs). These multi-input synapses have been suggested to store memory when LTP is blocked [15, 16].

Here, we studied whether and how the synaptic basis of hippocampal memory changes with age, using ultrastructural and molecular analyses after contextual fear conditioning (CFC) in young and aged mice.

## Results

### Impaired Memory Destabilization/Reconsolidation in Aged Mice

**Figure 1:**
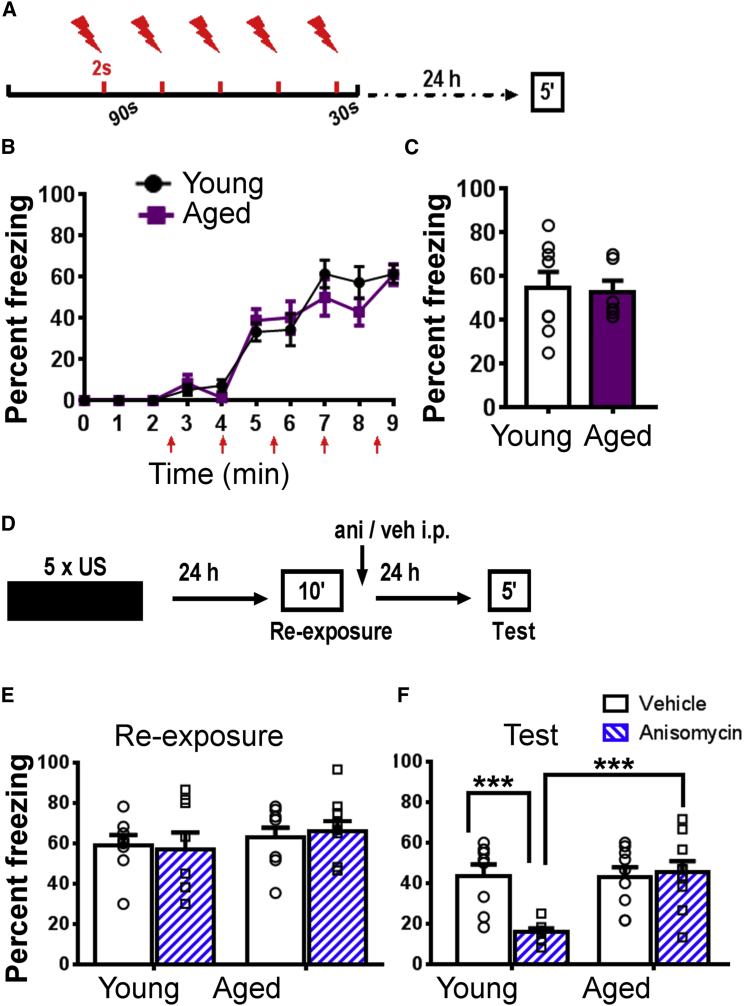
Aged Mice Can Form Contextual Fear Memory as Young Mice, but Reconsolidation of Their Memory Is Impaired (A) The CFC protocol is schematically shown. After 148 s, a 2-s foot shock (0.70 mA) was given and followed by four more foot shocks in 90-s intervals. Thirty seconds after the last foot shock the mouse was returned to its home cage and tested for contextual memory 24 h after conditioning. (B) Young (n = 8) and aged mice (n = 6) did not differ in CFC, as shown by the relative time spent freezing (effect of aging, F_(1,125)_ = 0.22, p = 0.65; effect of training, F_(8,125)_ = 60.6, p < 0.001; interaction age x training, F_(8,125)_ = 1.22, p = 0.30). (C) Aged mice had the same level of contextual freezing 24 h after CFC as young mice (unpaired t test, p = 0.93). (D) Experimental design for testing whether contextual fear memory is destabilized by retrieval, the initial step of reconsolidation which is thought to be required for memory updating [21]. Immediately after re-exposure to the context the protein synthesis blocker anisomycin (Ani; 225 mg/kg) or vehicle (veh; saline) was injected intraperitoneally (i.p.) and after 24 h contextual fear memory was re-tested (test). (E) Both young (n_Yveh =_ 8, n_YAni_ = 8) and aged mice (n_Aveh_ = 9, n_AAni_ = 10) had similar contextual fear memory during re-exposure. Note that the vehicle or anisomycin treatment occurred after re-exposure. (F) Anisomycin treatment (open bars versus filled bars; circles and rectangles show individual data plots for vehicle and anisomycin treatment, respectively) did not destabilize contextual fear memory in aged mice in contrast to young mice (effect of age, F_(1,31)_ = 8.53, p < 0.01; effect of treatment, F_(1,31)_ = 6.49, p < 0.01; interaction treatment x age, F_(1,31)_ = 9.22, p < 0.01; Tukey’s post hoc tests: Y_veh_ versus Y_Ani_, p < 0.001; A_veh_ versus A_Ani_, p = 0.72; Y_veh_ versus A_veh_, p = 0.94; Y_Ani_ versus A_Ani_, p < 0.001). Mean ± SEM, ^∗∗∗^p < 0.001. Individual data plots representing each animal within the group overlay the bar graphs.

To compare memory storage in young and aged mice, we chose CFC as the behavioral paradigm, where the animals learn to associate a novel environment with an aversive stimulus (foot shock). Even after strong CFC, using multiple foot shocks in one conditioning session, the task is hippocampus dependent [15, 17]. Mechanisms of memory storage are most adequately studied when acquisition of behavioral long-term memory is successful. Whereas weak CFC is impaired in aged mice [18, 19], we found that strong CFC with young (4 month old) and aged mice (18 month old) resulted in the same acquisition and 24-h memory retention in young (4 month old) and aged mice (18 month old) when freezing was measured (Figures 1A–1C). Twenty-four-hour contextual fear memory is considered to be a consolidated long-term memory [20]. Because young and aged mice acquired contextual fear memory at the behavioral level, we further assessed whether at both ages this acquired memory is of similar flexibility. To this end, we studied retrieval-induced memory reconsolidation that allows for memory updating in young animals [21]. Reconsolidation consists of destabilization and restabilization. The gold standard for assessing memory reconsolidation is the use of an amnestic agent immediately after memory retrieval, impairing restabilization [21]. Therefore, immediately after retrieval induced by re-exposure to the context, restabilization of contextual fear memory was pharmacologically blocked by systemic administration of anisomycin to test for memory destabilization (Figures 1D–1F). Further, we used a 10-min re-exposure, the strongest known protocol to induce destabilization of contextual fear memory [22]. As expected, we found that in young mice, anisomycin treatment impaired contextual fear memory after retrieval, demonstrating memory destabilization. However, in aged mice, post-retrieval memory was not blocked by anisomycin, showing impaired memory destabilization in aged mice. Impaired memory destabilization is thought to be due to distinct memory encoding [23]. Therefore, our findings suggested that the mechanistic basis of contextual fear memory may differ with age (see [23]).

### In Young but Not Aged Mice, Contextual Fear Memory Formation Is Associated with Ultrastructural and Molecular Alterations Underlying LTP

**Figure 2:**
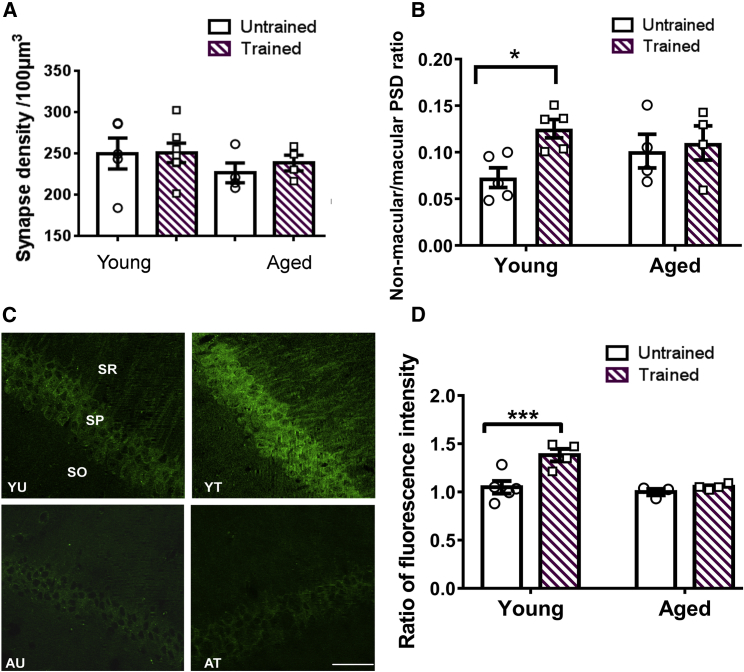
In Young but Not Aged Mice, Contextual Fear Memory Formation Is Associated with Ultrastructural and Molecular Alterations Characteristic for LTP (A) In young and aged mice, excitatory synapse density did not change 24 h after CFC as assessed by EM analysis (n_YU_ = 5, n_YT_ = 7, n_AU_ = 4, n_AT_ = 4; effect of age, F_(1,16)_ = 1.55, p = 0.23; effect of CFC, F_(1,16)_ = 0.21, p = 0.65; interaction CFC x age, F_(1,16)_ = 0.16, p = 0.70). (B) In young but not aged mice, contextual fear memory formation was associated with an increase in non-macular, complex PSDs at the expense of macular PSD (n_YU_ = 5, n_YT_ = 5, n_AU_ = 4, n_AT_ = 4; ratio non-macular to macular PSDs: effect of age, F_(1,14)_ = 0.22, p = 0.65; effect of training, F_(1,14)_ = 4.80, p < 0.05; interaction CFC x age, F_(1,14)_ = 2.44, p = 0.14; Tukey’s post hoc tests, YU versus YT, p < 0.05; AU versus AT, p = 0.68). (C) Representative images of T286-autophosphorylated αCaMKII in dorsal CA1 stratum radiatum (SR), stratum pyramidale (SP), and stratum oriens (SO) in young and aged mice before and 2 h after CFC. The fluorescence intensity in SR was referred to fluorescence intensity in SO (see STAR Methods). The scale bar represents 20 μm. (D) Quantification of the immunohistochemistry showed that contextual fear memory formation induces T286-autophosphorylation in young but not in aged mice (n_YU_ = 5, n_YT_ = 4, n_AU_ = 3, n_AT_ = 4; effect of age, F_(1,12)_ = 11.3, p = 0.06; effect of CFC, F_(1,12)_ = 12.02, p < 0.01; interaction CFC x age, F_(1,12)_ = 6.13, p < 0.05; Tukey’s post hoc tests: YU versus YT, p < 0.001; AU versus AT, p = 0.52). Mean ± SEM, ^∗∗∗^p < 0.001, ^∗^p < 0.05. Individual data plots representing each animal within the group overlay the bar graphs. See also Figures S1–S4.

NMDA receptor-dependent LTP at synapses in the stratum radiatum of hippocampal area CA1 is thought to underlie contextual fear memory formation in young animals [24, 25, 26]. Even though aging causes a shift from NMDA receptor- to voltage-gated Ca^2+^ channel (VGCC)-dependent LTP, which is associated with an increased threshold of LTP induction [10, 12, 14], inhibition of VGCCs facilitates hippocampal memory formation in aged animals [27, 28, 29, 30, 31]. This suggests that NMDA receptor-dependent LTP also enables hippocampal memory formation in aged animals, as in young ones [12]. We tested this idea after strong CFC (Figures 1A–1C) by analyzing ultrastructural and molecular alterations underlying LTP in young and aged mice. Using serial electron microscopy (EM), we 3-dimensionally (3D) analyzed about 2,000 synapses per animal in the stratum radiatum of hippocampal area CA1, which receives Schaffer collateral input, before and 24 h after CFC. We found that the density of excitatory and inhibitory synapses in CA1 stratum radiatum did not change after CFC in young or aged mice (Figures 2A, S1, and S2). However, when considering synapse and PSD morphology, we found that in young mice, contextual fear memory formation was associated with an increase in non-macular, complex PSDs at the expense of simple macular PSDs (Figure 2B), an increase in mushroom-type spines, as well as a decrease in thin spines (Figure S3). Both the increase in non-macular, complex PSDs and mushroom spines is characteristic of structural LTP [5, 6, 7, 32]. This significant increase in mushroom spines after CFC in young mice (Figure S3; Table S1) is also consistent with an earlier study showing a specific 40% increase in mushroom spines expressing a GluA1 reporter protein after CFC in young mice [26].

However, unlike young mice, aged mice did not show an increase in non-macular, complex PSDs and mushroom-type spines (Figures 2B and S3; Table S1), or any other change in synapse number and morphology. Therefore, the aged mice lacked the structural correlate of LTP, although they formed contextual fear memory similar to young mice (Figures 1C and 1E).

Next, we analyzed the T286-autophosphorylation of αCaMKII, which is essential for the induction of NMDA receptor-dependent LTP in CA1 stratum radiatum [33]. This is detectable with immunohistochemistry [34] and lasts for several hours after LTP induction [35]. We studied the T286-autophosphorylation before and 2 h after CFC, using immunohistochemistry (Figure 2C). In young mice, contextual fear memory formation was associated with an increase in T286-autophosphorylation in CA1 stratum radiatum (Figure 2D), while total αCaMKII levels did not change (Figure S4). This finding further supports our earlier suggestion that an LTP-like synaptic strengthening underlies contextual fear memory formation in young mice.

However, in aged mice, T286-autophosphorylation did not increase in CA1 stratum radiatum after CFC (Figures 2D and S4). The analysis of T286-autophosphorylation is consistent with our finding that structural LTP does not underlie contextual fear memory formation in aged mice (Figures 2B and S3).

### In Aged Mice, Contextual Fear Memory Formation Is Associated with Generation of Multi-innervated Dendritic Spines

**Figure 3:**
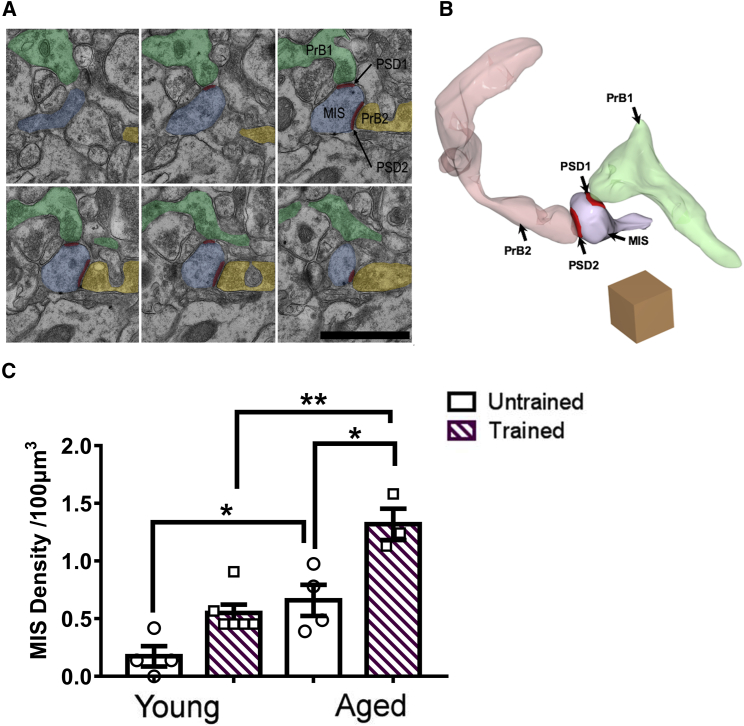
In Aged Mice, Contextual Fear Memory Formation Is Associated with Multi-innervated Dendritic Spine Generation (A) Serial EM showing a single MIS innervated (blue) by two presynaptic boutons (pre B1, green; pre B2, gold). The scale bar represents 1 μm. (B) Reconstructed EM images of MISs showing two independent postsynaptic densities (red) innervated by two presynaptic boutons originating from different axons (pink and green). The cube represents 1 μm^3^. (C) In aged but not young mice, contextual memory formation was associated with an increase of MIS density in CA1 SR (n_YU_ = 4, n_YT_ = 6, n_AU_ = 4, n_AT_ = 3; effect of age, F_(1,13)_ = 14.3, p < 0.01; effect of CFC, F_(1,13)_ = 7.35, p < 0.05; interaction CFC x age, F_(1,13)_ = 0.44, p = 0.51; Tukey’s post hoc tests: YU versus YT, p = 0.13; AU versus AT, p < 0.05; YT versus AT, p < 0.01). Additionally, MIS density was higher in AU than YU (p < 0.05). Individual data plots representing each animal within the group overlay the bar graphs. See also Video S1.

Work with T286-autophosphorylation-deficient mutant mice has suggested that generation of MISs can enable contextual fear memory formation when LTP is blocked [15, 16]. MISs are atypical excitatory synapses where a postsynaptic spine is innervated by two, rarely three, presynaptic boutons [36] (Figures 3A and 3B; Video S1). Using 3D EM, we analyzed 2,000 synapses per animal in CA1 stratum radiatum before and 24 h after CFC. Interestingly, aging increased MIS density in untrained mice (Figure 3C). Consistent with earlier work [15], CFC did not significantly increase MIS density in young mice (Figure 3C). In contrast, in aged mice, contextual fear memory formation was associated with a profound increase in MIS density (Figure 3C). We estimate that several million MISs could be generated in the total volume of CA1 stratum radiatum after CFC in aged mice (Table S2). These findings suggest that MIS generation may enable contextual fear memory formation in aged mice.

Video S1. Multi-innervated Dendritic Spine Stack at a Scale of 0.5 μm, Related to Figure 3

**Figure 4:**
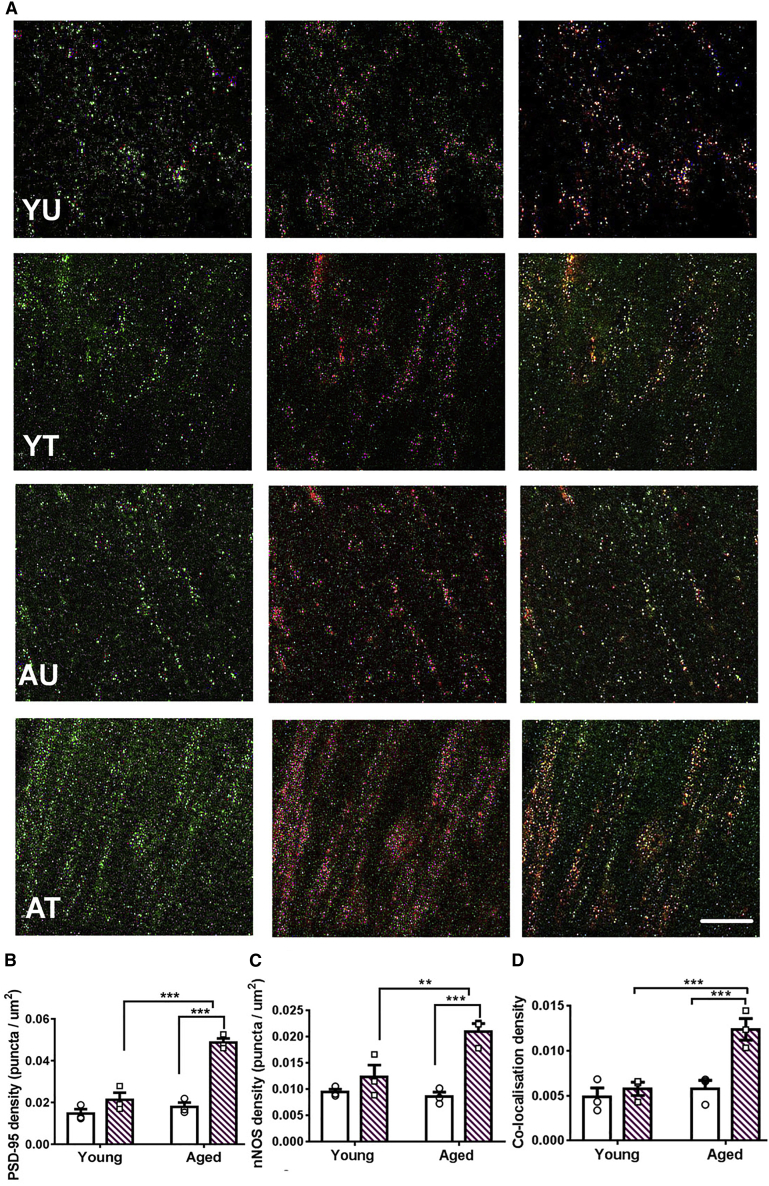
In Aged Mice, Contextual Fear Memory Formation Is Associated with Upregulation of PSD-95 and nNOS (A) PSD-95 upregulation is sufficient to induce MIS generation [37]. Representative images of separate co-immunolabeling experiments with PSD-95 (green) and neuronal nitric oxide synthase (nNOS; red) and merged images. The scale bar represents 20 μm. (B) Contextual fear memory formation in aged but not young mice was associated with PSD-95 upregulation (n = 3 each; effect of age, F_(1,8)_ = 58.85, p < 0.0001; effect of CFC, F_(1,8)_ = 39.41, p < 0.001; interaction CFC x age, F_(1,8)_ = 24.42, p = 0.001; Tukey’s post hoc tests: AU versus AT, p < 0.0001; YT versus AT, p = 0.0002). (C) Contextual fear memory formation in aged but not young mice was associated with nNOS upregulation (n = 3 each; effect of age, F_(1,8)_ = 8.16, p < 0.05; effect of CFC, F_(1,8)_ = 24.2, p < 0.001; interaction CFC x age, F_(1,8)_ = 7.22, p < 0.05; Tukey’s post hoc tests: YU versus YT, p = 0.15; AU versus AT, p < 0.001). (D) Contextual fear memory formation in aged but not young mice was associated with increased co-localization of PSD-95 and nNOS (n = 3 each; effect of age, F_(1,8)_ = 24.1, p < 0.001; effect of CFC, F_(1,8)_ = 32.0, p < 0.001; interaction CFC x age, F_(1,8)_ = 14.9, p < 0.001; Tukey’s post hoc tests: YU versus YT, p = 0.21; AU versus AT, p < 0.001). Mean ± SEM, ^∗∗∗^p < 0.001, ^∗∗^p < 0.01, ^∗^p < 0.05. Individual data plots representing each animal within the group overlay the bar graphs. See also Figure S7.

MISs develop by the attraction of presynaptic terminals onto existing synapses [36, 37, 38, 39]. *In vitro* MIS generation is caused by increased PSD-95 expression followed by PSD-95 interaction with neuronal nitric oxide synthase (nNOS) and resulting nitric oxide signaling [38]. Because MIS generation is associated with contextual fear memory formation in aged, but not young mice (Figure 3C), we tested whether this is linked with underlying molecular mechanisms [16, 38]. Sequential immunolabeling of PSD-95 and nNOS in CA1 stratum radiatum was performed (Figure 4). The density of PSD-95 puncta was upregulated after CFC only in aged but not young mice, seen in two independent experiments (Figures 4A, 4B, and S5). The majority of nNOS puncta co-localized with PSD-95, consistent with a postsynaptic enrichment of nNOS in CA1 stratum radiatum [40]. Furthermore, the density of nNOS puncta and the co-localization of PSD-95 and nNOS were upregulated after CFC in aged, but not young mice (Figures 4A, 4C, and 4D). Although we did not study PSD-95 expression with sparse labeling of spines, the vast majority of PSD-95 expression is known to be in spines. Therefore, the substantial PSD-95 upregulation in the stratum radiatum after CFC in aged mice will affect spines. This molecular analysis is consistent with the finding that contextual fear memory is associated with MIS generation in aged, but not young mice (Figure 3C). It also demonstrates that aged mice engage molecular signaling mechanisms during contextual fear memory formation that differ from signaling in young mice (see also Figures 2C and 2D).

### Contextual Fear Memory Formation in Aged but Not Young Mice Is Blocked by ZL006

**Figure 5:**
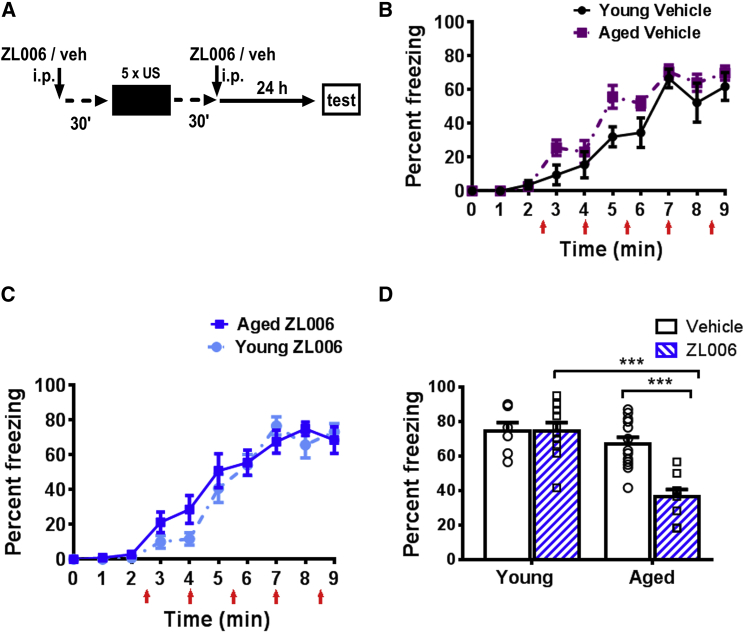
Contextual Fear Memory Formation in Aged but Not Young Mice Is Blocked by ZL006 (A) ZL006 (10 mg/kg, i.p.) or vehicle (veh) was administered 30 min before and 30 min after CFC, and contextual fear memory was assessed 24 h after CFC. (B) No effect on acquisition of contextual fear memory after vehicle injection in young and aged mice. (C) No effect on acquisiton by ZL006 treatment in young and aged mice (n_Yveh =_ 7, n_YZL006_ = 10; effect of treatment, F_(1,120)_ = 1.2, p = 0.30; effect of training, F_(8,120)_ = 75.0, p < 0.001; interaction treatment x training, F_(8,120)_ = 1.6, p = 0.15) and aged mice (n_Aveh_ = 13, n_AZL006_ = 9; effect of treatment, F_(1,160)_ = 0.029, p = 0.87; effect of training, F_(8,160)_ = 86.0, p < 0.001; interaction treatment x training, F_(8,160)_ = 0.71, p = 0.68). (D) Contextual fear memory 24 h after conditioning was impaired in ZL006-treated aged but not young mice (effect of age, F_(1,35)_ = 24.6, p < 0.001; effect of treatment, F_(1,35)_ = 11.1, p < 0.01; interaction age x treatment, F_(1,35)_ = 10.9, p < 0.01; Tukey’s post hoc tests: Y_veh_ versus Y_ZL006_, p = 0.99; A_veh_ versus A_ZL006_, p < 0.001; Y_ZL006_ versus A_ZL006_, p < 0.001). Mean ± SEM, ^∗∗∗^p < 0.001. Individual data plots representing each animal within the group overlay the bar graphs. See also Figures S5 and S6.

To elucidate the importance of the age-specific molecular changes (Figures 4 and S5) for contextual fear memory formation, we used ZL006, a compound that inhibits the interaction of PSD-95 with nNOS [41]. Previous studies have shown that ZL006 does not affect spatial memory formation, locomotion, motor function, or motivation in young rodents [41, 42, 43], but the compound has not previously been tested on aged animals. We tested the impact of ZL006 treatment (10 mg/kg, intraperitoneally; i.p.) on contextual fear memory formation in young and aged mice (Figures 5A–5C). After systemic administration, it takes about 1 h until ZL006 levels are maximal in the brain [41]. Thus, we administered ZL006 twice, 30 min before and 30 min after CFC, to keep ZL006 levels high during memory consolidation. The ZL006 treatment did not affect contextual fear memory acquisition in young and aged mice (Figure 5C). However, ZL006 treatment impaired 24-h contextual fear memory in aged but not young mice (Figure 5D). To our knowledge, this is the first time that a drug treatment has been shown to distinguish between memory formation in aged and young mice. Together, these findings suggest that ZL006 blocks consolidation of contextual fear memory in aged but not in young mice. We also found that ZL006 administration 30 min after CFC did not impair contextual fear memory formation in aged mice (Figure S6), suggesting that the PSD-95-nNOS interaction is not required for a late consolidation process.

**Figure 6:**
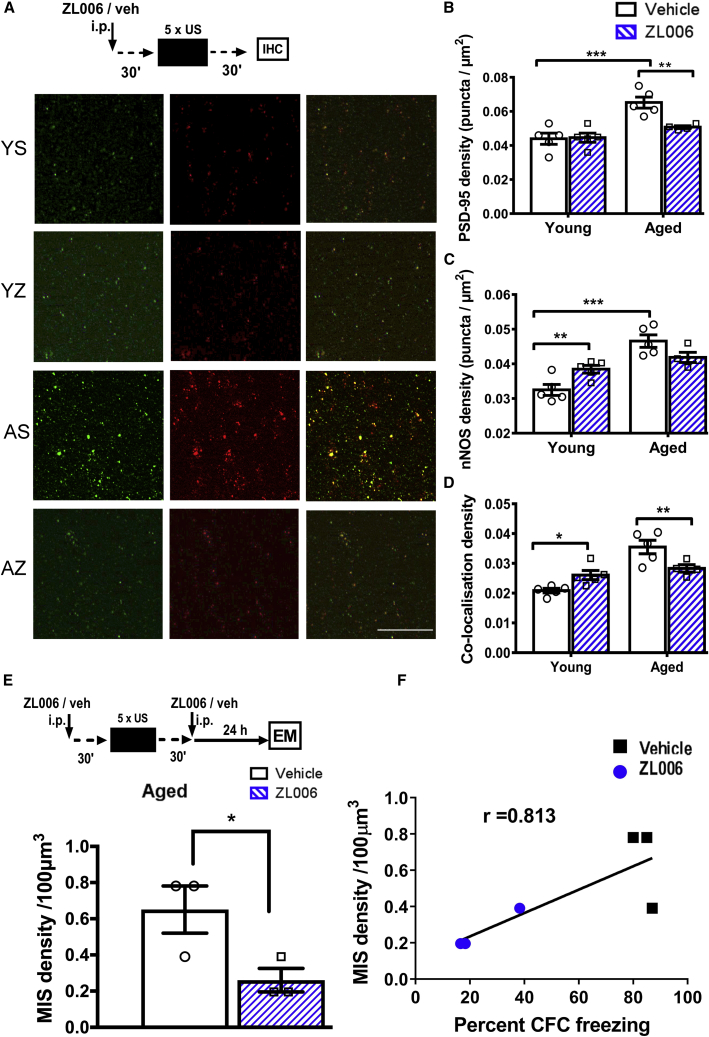
ZL006 Blocked PSD-95-nNOS-Mediated Upregulation of MISs in Aged Mice (A) Experimental design to study the impact of ZL006 treatment on PSD-95 and nNOS and representative images showing PSD-95 (green) and nNOS (red) puncta and merged images in dorsal CA1 stratum radiatum. The scale bar represents 10 μm. n_Yveh =_ 5, n_YZL006_ = 5, n_Aveh_ = 5, n_AZL006_ = 4. (B) ZL006 significantly reduced PSD-95 expression in conditioned, aged, but not in conditioned, young mice (effect of age, F_(1,15)_ = 21.5, p < 0.001; effect of treatment, F_(1,15)_ = 5.90, p < 0.05; interaction age x treatment, F_(1,15)_ = 6.2, p < 0.05; Tukey’s post hoc tests: AT_veh_ versus AT_ZL006_, p < 0.01; YT_veh_ versus YT_ZL006_, p < 0.001). (C) There was a statistical trend that ZL006 treatment reduced nNOS levels in aged mice (effect of age, F_(1,15)_ = 32.0, p < 0.001; effect of treatment, F_(1,15)_ = 0.17, p = 0.68; interaction age x treatment, F_(1,15)_ = 12.0, p < 0.01; Tukey’s post hoc tests: YT_veh_ versus YT_ZL006_, p < 0.01; AT_veh_ versus AT_ZL006_, p = 0.053; YT_veh_ versus AT_veh_, p < 0.001). (D) ZL006 reduced co-localization of PSD-95 and nNOS in conditioned, aged mice, whereas it surprisingly increased the co-localization in conditioned, young mice (n = 5 each, except n_AZL006_ = 4; effect of age, F_(1,15)_ = 27.9, p < 0.001; effect of treatment, F_(1,15)_ = 0.38, p = 0.54; interaction age x treatment, F_(1,15)_ = 15, p < 0.01; Tukey’s post hoc tests: YT_veh_ versus AT_veh_, p < 0.001; YT_veh_ versus YT_ZL006_, p < 0.05; AT_veh_ versus AT_ZL006_, p < 0.01). (E) ZL006 (10 mg/kg, i.p.) was administered 30 min before and 30 min after CFC, and 24 h after CFC an EM analysis was carried out. ZL006 treatment significantly reduced MIS density in aged mice 24 h after CFC (n_veh_ = 3, n_ZL006_ = 3; one-tailed t test, t(4) = 2.68, p < 0.05). (F) Scatterplot showing a positive correlation between freezing and MIS density 24 h after CFC. Data are from aged, ZL006-treated mice (blue circles) and aged, vehicle-injected mice (black squares) (n = 3 each; r = 0.813, p = 0.048, Pearson correlation). Mean ± SEM, ^∗∗∗^p < 0.001, ^∗∗^p < 0.01, ^∗^p < 0.05. Individual data plots representing each animal within the group overlay the bar graphs. See also Figures S6 and S7.

We examined the effect of ZL006 treatment on the density of PSD-95 and nNOS, and their co-localization, 30 min after CFC (Figures 6A–6D). In aged mice, ZL006 treatment reduced the PSD-95 and nNOS density and co-localization to the same level as in young mice (Figures 6B–6D). Thus, ZL006 treatment had the expected impact in aged mice after CFC. Interestingly, the ZL006 treatment also blocked PSD-95 upregulation in aged mice 30 min after CFC (Figure 6B). Accordingly, PSD-95 upregulation after CFC in aged mice (Figure 4) might be downstream of signaling triggered by the interaction of PSD-95 with nNOS. We also assessed the impact of ZL006 treatment on synapses in CA1 stratum radiatum in aged mice 24 h after CFC (Figures 6E and S6), the time point when contextual fear memory was tested (Figure 5C). Our data analysis indicates that ZL006 treatment reduced MIS density in the trained, aged mice (Figure 6E). Furthermore, the ZL006/saline experiment established a positive correlation between MIS density in CA1 stratum radiatum and 24-h contextual freezing (Figure 6F). Therefore, we suggest that MIS generation contributes to contextual fear memory formation in aged mice.

## Discussion

**Figure 7:**
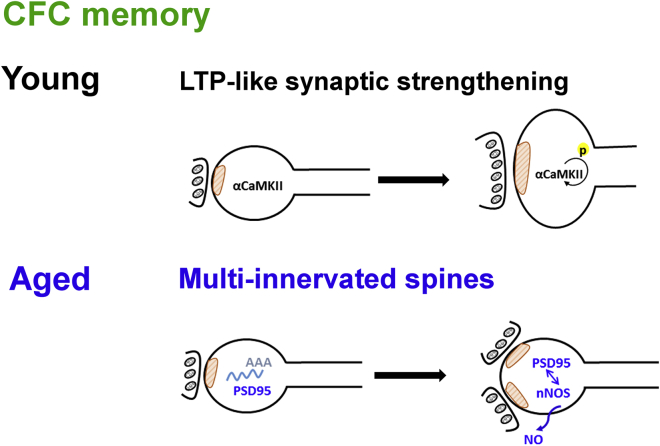
Model for Distinct Memory Mechanisms in Young and Aged Mice In young mice, memory appears to underlie an LTP-like synaptic strengthening that involves the autophosphorylation of CaMKII and structural changes at existing synapses. In aged mice, memory appears to underlie MIS generation caused by nitric oxide retrograde signaling due to PSD-95-nNOS interaction.

Here, we provide evidence that the synaptic basis of hippocampal memory changes with age (Figure 7). Using a strong, hippocampus-dependent CFC paradigm [15, 17] that results in equal levels of behavioral contextual fear memory in young and aged mice, we identified differences at the ultrastructural and molecular levels in hippocampal area CA1. We also revealed for the first time a molecular manipulation that impairs memory formation in aged but not in young animals.

NMDA receptor-dependent LTP in hippocampal area CA1 is thought to underlie contextual fear memory formation in young animals [24, 25, 26]. In agreement with this earlier work, we also found evidence that LTP underlies contextual fear memory at a young age. Specifically, we detected a lasting, conditioning-induced increase in mushroom spines at the expense of thin spines, and an increase in non-macular, complex PSDs at the expense of macular PSDs, changes in synapse morphology that are characteristic for structural LTP [5, 6, 7, 32]. Furthermore, there was a conditioning-induced increase in the autophosphorylation of αCaMKII, a fundamental signaling step underlying NMDA receptor-dependent LTP [33, 44].

Although we detected an LTP-like strengthening of synapses after CFC in young mice, this was not observed in aged mice. Therefore, we conclude that aged mice do not use NMDA receptor-dependent LTP, or any other type of LTP, to store contextual fear memory. It is conceivable that in old age the increased threshold for LTP induction may not be reached by CFC to obtain LTP for memory storage, unlike with artificial, electric stimulation [10, 12, 14]. An alternative interpretation is that aged mice may induce only short-lasting, early LTP after CFC, as aging impairs the late phase of LTP [45]. The late phase of LTP requires synaptic tagging by αCaMKII [46] and aging leads to reduced αCaMKII activity due to direct oxidation of the protein [47]. The idea of an age-related impairment in synaptic tagging due to αCaMKII dysfunction is consistent with our finding that αCaMKII autophosphorylation is not increased 2 h after CFC in aged mice.

Our findings suggest that in aged but not in young mice, MIS generation may be critical for contextual fear memory. We observed for the first time that in the wild-type genotype, MIS generation can be associated with memory formation. MISs are a rare type of synapse. We found that aging elevates MIS density in CA1 stratum radiatum and, after CFC, MIS density increased further. Therefore, a sufficient number of MISs may be generated to store contextual fear memory (see Table S2), which is thought to involve only a few synapses, allowing for sparse coding.

MISs develop by the attraction of presynaptic terminals onto existing synapses, involving upregulation of PSD-95, interaction between PSD-95 and nNOS, and retrograde signaling by nitric oxide [36, 37, 38, 39]. We detected these underlying molecular processes during contextual fear memory formation in aged but not in young mice. Interestingly, the CFC-induced PSD-95 upregulation in aged mice does not result in an overall elevation of synapse numbers, which is consistent with PSD-95 overexpression studies in hippocampal slice cultures [38]. Further, the amount CFC-induced PSD-95 upregulation is much larger than MIS generation, indicating that there are more rate-limiting steps to generate MISs, such as PSD-95 and nNOS interaction, retrograde signaling, and additional molecular processes to establish the second presynaptic input onto an existing synapse.

We provide evidence that blocking the interaction between PSD-95 and nNOS inhibits MIS generation and memory formation specifically in aged mice. Furthermore, we found a positive correlation between MIS density and contextual freezing in aged mice. This suggests that MIS generation contributes to contextual fear memory formation in aged mice and poses the question: why can aged mice generate MISs but not LTP after CFC? MIS generation probably depends on NMDA-receptor activation [37]. Thus, downstream of NMDA-receptor activation there may be competition between LTP induction and MIS generation. Our previous work suggests that at a young age, αCaMKII signaling suppresses MIS generation [15, 16]. In old age, however, CaMKII activity is compromised due to oxidation of the protein [47], which may favor MIS generation at the expense of LTP. MIS generation leads to a long-lasting change in connectivity rather than a long-term increase in transmission at a synapse existing before learning.

Previous work has shown that weak CFC is impaired in aged mice [18, 19]. We suggest that in those studies, MISs could not be generated due to a lack of signaling above a threshold, as in αCaMKII T286-autophosphorylation-deficient mice contextual fear memory formation and PSD-95 upregulation occur only after strong but not weak CFC [15]. Therefore, strong training may be needed to induce sufficient signaling for MIS generation. In aged mice, the effect of extensive training in various hippocampus-dependent memory tasks has not been systemically studied, as far as we know. However, it is established that extended training in the Morris water maze enables spatial memory formation in aged mice (e.g., [14]). It remains to be tested whether this spatial memory formation in aged mice depends on MIS generation. Moreover, under some experimental conditions, it has been noted that some aged rodents can form memory whereas others cannot (e.g., [14, 48]). Follow-up investigations are needed to test whether memory formation in those aged animals is based on MIS generation.

Memory reconsolidation has been suggested to update memory storage [21, 23]. Reconsolidation involves initial destabilization followed by protein-synthesis-dependent restabilization. Destabilization can be analyzed when restabilization is blocked. To our knowledge, destabilization has only been studied at a young age. An earlier study suggested that reconsolidation is impaired in aged rats and humans [49]. However, this study did not block protein synthesis to assess memory destabilization. Here, we show that memory destabilization is impaired in aged mice. We detected this impairment using a re-exposure protocol that induces destabilization of strong contextual fear memory in young mice. Thus, in old age, memory destabilization may not only be impaired, it may be completely blocked. Previous work on boundary conditions for inducing destabilization at a young age indicated that strong encoding prevents destabilization [23]. Thus, it is conceivable that impaired memory destabilization in aging is due to the involvement of MISs, as the reversal of these multi-input synapses into one-input synapses might not be induced by retrieval. This idea is based on the observation that in dual spines the excitatory synapse is unusually stable [50]. Thus, an MIS-based memory-storing mechanism may explain why memory updating, a fundamental cognitive process [21], is impaired in old age [49, 51].

## STAR★Methods

### Key Resources Table

**Table:** 

REAGENT or RESOURCE	SOURCE	IDENTIFIER
**Antibodies**		

Anti-CaMKII (total)	Millipore	Cat# MAB8699; RRID: AB_2067919
Anti-CaMKIIalpha (phospho T286)	Abcam	Cat# AB5683; RRID: AB_305050
Anti-PSD-95	Abcam	Cat# AB18258; RRID: AB_444362
Anti-nNOS (neuronal nitric oxide synthase)	Abcam	Cat# AB1376; RRID: AB_300614

**Chemicals and Drugs**		

ZL006	Sigma Aldrich	Cat# SML0146
Anisomycin	Sigma Aldrich	Cat# A9789
DMSO	Sigma Aldrich	Cat# 276855
0.9% Normal saline	Sigma Aldrich	Cat# S8776

**Experimental Model: Animals**		

Mouse-C57BL/6J	Harlan,NL	Strain Code: C57BL/6JRccHsd:

**Softwares and Algorithms**		

Fiji	ImageJ	https://imagej.net/Fiji/Downloads
Prism 6	GraphPad software	https://www.graphpad.com/scientific-software/prism/
Sigma Plot	Systat software	https://systatsoftware.com/sp/download.html
Photomerge™ CS6	Adobe Photoshop CS6	https://www.adobe.com/uk
SEM. Align 1.26b program	1.26b program	http://synapseweb.clm.utexas.edu/
Reconstruct	Synapse web	http://synapseweb.clm.utexas.edu/software-0
3D Studio Max	Studio Max 2016	https://www.autodesk.eu

### Lead Contact and Materials Availability

Further information and requests for resources and reagents should be directed to the Lead Contact, Karl Peter Giese (karl.giese@kcl.ac.uk).

This study did not generate unique new reagents.

### Experimental Model and Subject Details

Experiments were conducted using female C57BL/6J inbred mice (Harlan, NL), aged mice were 18-22 months and young mice 3-4 months old. Mice were group housed under standard laboratory conditions with food and water *ad libitum*. All work involving mice was conducted in accordance with the UK Animals Scientific Procedures Act 1986 and was approved by Animal facility of IOPPN, King’s College London.

### Method Details

#### Contextual fear conditioning (CFC) and memory reconsolidation experiment

The mice were trained in a conditioning chamber (Med Associates) in a sound attenuating box with background noise supplied to the chamber by a white noise generator as described [15]. Each mouse was placed in the chamber and was presented with a foot shock (2 s, 0.7 mA) after an introductory period of 148 s. The shock was repeated four times with an inter-trial interval of 90 s. Each mouse was returned to its home cage 30 s after presentation of the last shock. Contextual fear memory was tested 24 h after training by re-exposure to the conditioning chamber for 5 min. A video camera was fixed inside the door of the sound attenuating chamber, allowing the behavior to be observed and scored. Freezing behavior (defined as a complete lack of voluntary movement) was scored for 2 s in every 5 s. All procedures were done double blind to experimental treatment.

For memory reconsolidation experiments, contextual fear memory was reactivated 24 h after training by re-exposing the mouse to the conditioning chamber for 10 min, the known maximal re-exposure time to induce memory reconsolidation [22]. The animals received anisomycin injections, immediately after memory reactivation. Anisomycin (Sigma-Aldrich) was dissolved in DMSO and normal saline 0.9% (1:5) and injected intraperitoneally (i.p.) at a dose of 225 mg/kg. One day after the re-exposure contextual fear memory was assessed in the conditioning chamber for 5 min. Two aged animals that showed cloudy eyes and restricted mobility after anisomycin injection were excluded from further experimentation.

#### ZL006 experiments

ZL006 (Sigma-Aldrich) was dissolved in DMSO and normal saline (1:4). Experiments were carried out double blind. Mice were randomly assigned to vehicle or ZL006 group. Mice were injected i.p. with ZL006 (10 mg/kg) or vehicle 30 minutes before CFC experiment. Injection volume was 1 ml/kg and control animals were injected with an equal volume of vehicle. After 30 minutes they were taken to CFC chamber and conditioned with five footshocks. Thirty minutes after CFC mice were given another i.p. injection of either vehicle or ZL006 (10 mg/kg). Some animals were perfused for IHC immediately after the second injection of ZL006 or vehicle (Figures 6A–6D). Some animals were tested for contextual fear memory 24 hours after CFC (Figures 5, 6E, and 6F). Two aged mice (one saline and one ZL006-treated) were excluded from analysis, as they showed unusual freezing before shock presentation at training. Immediately after memory testing some animals were perfused for EM analysis (Figures 6E and 6F).

#### Immunohistochemistry

4% PFA was used for perfusion and fixation at different time points after CFC training. Coronal brain sections (40 μm thick) were prepared (Microm HM560) and stored at –20°C in PBSAF [PBS, 20% sucrose (Sigma-Aldrich), 15% ethylene glycol (Sigma-Aldrich), and 0.05% NaN_3_ (Sigma-Aldrich). Dorsal hippocampal brain slices starting at −1.5 mm of Bregma with every 3-6^th^ section were used for staining with different antibodies (Key Resources Table). Sequential staining was performed for PSD-95 and nNOS co-localization studies. Sections were incubated in primary antibody solution overnight at RT or 4°C. Slices were washed in PBS and incubated in secondary antibody solution for 2 h at RT. Slices were washed and mounted onto Polylysine slides (VWR, DE) with VectaShield® containing 4’,6-Diamidino-2-Phenylindole (DAPI) LOT: ZA0210 (Vector, US) as the mounting medium.

#### Light microscopy imaging and analysis

Immunostaining was analyzed with AxioImager Z1 microscope. Photomicrographs of stained brain sections were taken with a digital camera (AxioCam MRm, Zeiss). Six to eight Z stacks of microphotographs were taken per animal, from every sixth section of the dorsal hippocampus stratum radiatum of CA1 field. Z stacks were reconstructed to maximal projections and analyzed with ImageJ software. The TIFF format micrographs were analyzed with ImageJ software. The threshold tool was used, which identifies objects distinct from the background based on coloring and intensity. Every sixth section from the dorsal hippocampus, was analyzed for total αCaMKII and T286-autophosphorylated αCaMKII. αCaMKII density was calculated by taking measurements from three identical rectangular areas on images and taken as a ratio of mean background density using the corresponding mean CA1 stratum oriens (SO) αCaMKII background value for each animal [33]. The intensity of fluorescence did not change in SO before and after training in all mouse groups making it a reliable indicator for subtracting background autofluorescence.

Fluorescent immunostaining for PSD-95 and nNOS was analyzed with AxioImager Z1 microscope, with Plan-Apochromat 63 × objective and Apotome (Zeiss). Four Z stack micrographs (10 pictures per stack, every 0.5 μm) were taken with AxioCamMT3 M27 per brain section, and every sixth section through the dorsal hippocampus (stratum radiatum of CA1 field; Bregma −1.58 to −2.18 mm) was analyzed (on average five sections per animal). Z stacks were reconstructed with ImageJ software and processed with “find edges” and “max projection” functions. PSD-95 and nNOS immunostaining appeared as puncta. The density of PSD-95 and nNOS puncta, as well as their co-localization were analyzed and measured using the analyze particle tool (Fiji software). Briefly, the entire image field was processed for signal quantification and images were converted to grayscale (8 bits/pixel) and threshold tool was used, which identifies objects distinct from the background based on intensity. Puncta after thresholding appeared as isolated disc like structures making it distinct from diffuse background. Counts from multiple sections were done to produce average values for each mouse. Each channel underwent thresholding to remove leftover background and a polygon selection tool was used to highlight a region of interest (ROI) avoiding soma and clumps of antibody. Density of PSD-95 and nNOS positive dots was calculated with ImageJ software according to formula: dot number/area (density of puncta). This was repeated for the remaining channel using the same ROI. To assess co-localization density of PSD-95 and nNOS puncta, the two channels were overlapped and co-localized particles counted using the previous ROI.

To reliably take images from CA1 SR, a low magnification image was taken using DAPI counterstain that clearly demarcates SP, SR and SO layers in CA1 area (Figure S7). The dorsal hippocampus CA1 region was consistently imaged across all groups as shown in Figure S7. In low magnification images, dendritic pattern of staining for PSD-95 and nNOS was seen clearly in SR with SP as a landmark (Figure S7) before going for higher magnification of 63x.

Tissue from all samples within a batch was run in parallel using the same intensity threshold. Analysis was conducted double blind to sample identity on batches that had been processed together. PSD-95 density experiments were repeated at two different time points after CFC, showing similar results indicating the reliability of experiment.

#### Electron microscopy

Mice were perfused intracardially with 3% PFA and 0.5% glutaraldehyde in 0.1 M phosphate buffer (pH 7.4). Brains were vibratomed coronally into 50 μm thick slices. Slices containing dorsal hippocampus were processed for electron microscopy, as described previously [32, 52, 53]. Tissue was dehydrated in graded aqueous solutions of ethanol from 30 to 100% (each for 10 min) and then 100% acetone (three changes, each for 10 min). Specimens were infiltrated with a mixture of 50% epoxy resin (Epon 812/Araldite CY212 epoxy resins) and 50% pure acetone for 2 h at room temperature. Each slice was flat-embedded – first, the slice was placed on an Aclar film and air-dried shortly to allow acetone evaporation, then a drop of pure resin was placed onto the slice and finally covered by a second Aclar film – and polymerized for 48 h at 60°C. Each slice was covered with a gelatine capsule containing pure epoxy resin and polymerized for 48 h at 60°C. The blocks with tissue slices were coded and all further analyses were carried out with the investigator blind to the experimental status of the tissue. The embedded slices on the block surface were trimmed with a glass knife along the entire surface of the hippocampus. A trapezoid area was prepared with a glass knife, which included both the CA1 pyramidal cells and CA1 stratum radiatum hippocampal layers as previously described [54]. Ultrathin serial sections (≥120 sections per tissue block) of gray/white color (60–70 nm) were cut with a Diatome diamond knife, allowed to form a ribbon on the surface of water in the knife bath and collected using slot copper grids with a carbon coated Pioloform film on it. Sections were counterstained with 3.5% aqueous uranyl acetate followed by Reynolds’ lead citrate. To allow uniform orientation of sections on adjacent grids during imaging in the electron microscope, a rotating grid holder was used. Serial sections were imaged in a JEOL JEM1400 electron microscope using an AMT XR60 camera in montaging mode at a column magnification of 6000x. Area of 18 μm x 11 μm (3x3 montage) was imaged in the middle of CA1 stratum radiatum. To determine middle area of CA1 stratum radiatum on serial ultrathin sections in EM we used similar approach used on brain sections impregnated by Golgi method. In CA1 nearly 50% of the dendritic length is located in stratum radiatum and 18% in stratum lacunosum-moleculare (SLM) [55]. For each animal we measured distance from CA1 pyramidal cell layer to hippocampal fissure, calculated the height of stratum radiatum and used the following formula to move from CA1 pyramidal cell layer to be in the middle of stratum radiatum: A = B^∗^0.74/2, where A = distance from CA1 pyramids to middle of stratum radiatum, B = distance from CA1 pyramidal cell layer to hippocampal fissure, 0.74 = percentage of stratum radiatum in total length from CA1 pyramidal cell layer to hippocampal fissure (sum of 50% and 18% was taken as 100% and proportion of stratum radiatum there was calculated as 74%). For example, if CA1 pyramidal cell layer-fissure distance was 340 μm, the distance from CA1 pyramid cell layer to reach middle of stratum radiatum was 126 μm.

#### Stereology and 3D EM reconstruction analysis

For stitching frames into montages Photomerge command in Adobe Photoshop CS6 was used and to automate the process for batch processing, a custom script was written in Adobe ExtendScript Toolkit CS6. Then montages were aligned to each other manually in SEM. Align 1.26b program (http://synapseweb.clm.utexas.edu/). After section alignment was finalized, stereological analysis of synapses and reconstruction of dendritic spines and their PSDs was done using Reconstruct software (http://synapseweb.clm.utexas.edu/software-0). Thickness of the sections was determined by the cylindrical diameters method [53].

Synapses were defined as contacts between neurons containing at least one PSD with a spine being the part of the synapse that contains PSDs, and a pre-synapse being the part of the synapse containing at least two to three vesicles. Additionally, MIS were classified, where two or more pre-synaptic boutons originated from different axons forming synapses with only one dendritic spine (Figures 3A and 3B).

The unbiased brick method [54, 56, 57, 58] was used for stereological analysis of total synaptic density. The series was evenly divided into 4 bricks (16 × 8 μm), 20 sections each with 10 sections gap between bricks. Three sides of the brick were inclusion planes, and the other three sides were exclusion planes. Those synapses (here we used postsynaptic density as a synapse marker) that were completely inside the brick or touching only inclusion planes were counted, whereas synapses touching the exclusion planes anywhere, were excluded. Each brick was divided into 2x2 μm squires to make actual counting easier. The density [Nv_syn_] per cubic micron of particular type of synapse (excitatory synapses, inhibitory synapses, and MIS) was estimated in each brick using the following formula: N_Vsyn_ = ΣQ^–^/h^∗^A (Q^–^ = total number of synapse type in the brick; h = height of the brick equal to sum of sections thicknesses included in the brick, in μm); A = area of the brick in μm^2^).

The results of 4 bricks were averaged; total number of synapses analyzed was approximately 2000 per series. Volumes of dendritic spines and postsynaptic densities were analyzed as follows. In each of four bricks 2 squares out of 32 were chosen by a random number generator between numbers 1 and 32. In chosen squares all countable (according to the unbiased brick rule, see above) dendritic spines and their PSDs were reconstructed. Total number of spines/PSDs reconstructed was ∼120 per animal. For frequency distribution analysis dendritic spines were categorized according to postsynaptic density volume. In our dataset PSD volume of 0.0032 μm^3^ corresponded to spine head diameter of 0.6 μm which is the biggest size for thin spines [59]. We found that spines with PSD volume between 0.0032 μm^3^ and 0.0044 μm^3^ didn’t have a spine apparatus and complex PSD, main features of mushroom spines. So we classified this group of spines as ‘intermediate’. Spines with a PSD > 0.0044 μm^3^ were classified as mushroom spines.

Individual synapses were identified as macular if the PSD profiles were continuous or as non-macular (perforated) if electron-lucent regions divided the PSD on adjacent serial sections, as described [60].

3D reconstructions of selected MIS were imported to 3D Studio Max 2016 software for rendering and subsequent rotation to display the optimal views of the reconstructed structures.

### Quantification and Statistical Analysis

All of the data are expressed as mean ± SEM “n” indicates number of animals. Shapiro–Wilk’s W test was applied for checking the normality of the datasets. Statistical significance was analyzed by one- or two-tailed unpaired Student t test for comparison between two groups. Two-way ANOVA was used for statistical comparison among multiple groups with drug treatments. Tukey’s post hoc test was further applied to allow the comparison of the mean of each group with the mean of the control group. Statistical significance was defined as p < 0.05. Statistical tests were carried out on SigmaPlot v13.0 or Prism.

### Data and Code Availability

This study did not generate/analyze datasets and code.

## Supplemental Information

Document S1. Figures S1–S7 and Tables S1 and S2

Document S2. Article plus Supplemental Information
