# Supplementary material for: Multi-input Synapses, but Not LTP-Strengthened Synapses, Correlate with Hippocampal Memory Storage in Aged Mice
Source: Curr Biol. 2019 Nov 4;29(21):3600–3610.e4. doi: 10.1016/j.cub.2019.08.064 (PMC6839404; doi:10.1016/j.cub.2019.08.064)
Supplement: Document S1. Figures S1–S7 and Tables S1 and S2 [file mmc1.pdf]

Current Biology, Volume *29*

Supplemental Information

Multi-input Synapses, but Not LTP-Strengthened Synapses, Correlate with Hippocampal Memory Storage in Aged Mice

Wajeeha Aziz, Igor Kraev, Keiko Mizuno, Alastair Kirby, Ton Fang, Huzefa Rupawala, Kamillia Kasbi, Stephanie Rothe, Felix Jozsa, Kobi Rosenblum, Michael G. Stewart, and K. Peter Giese

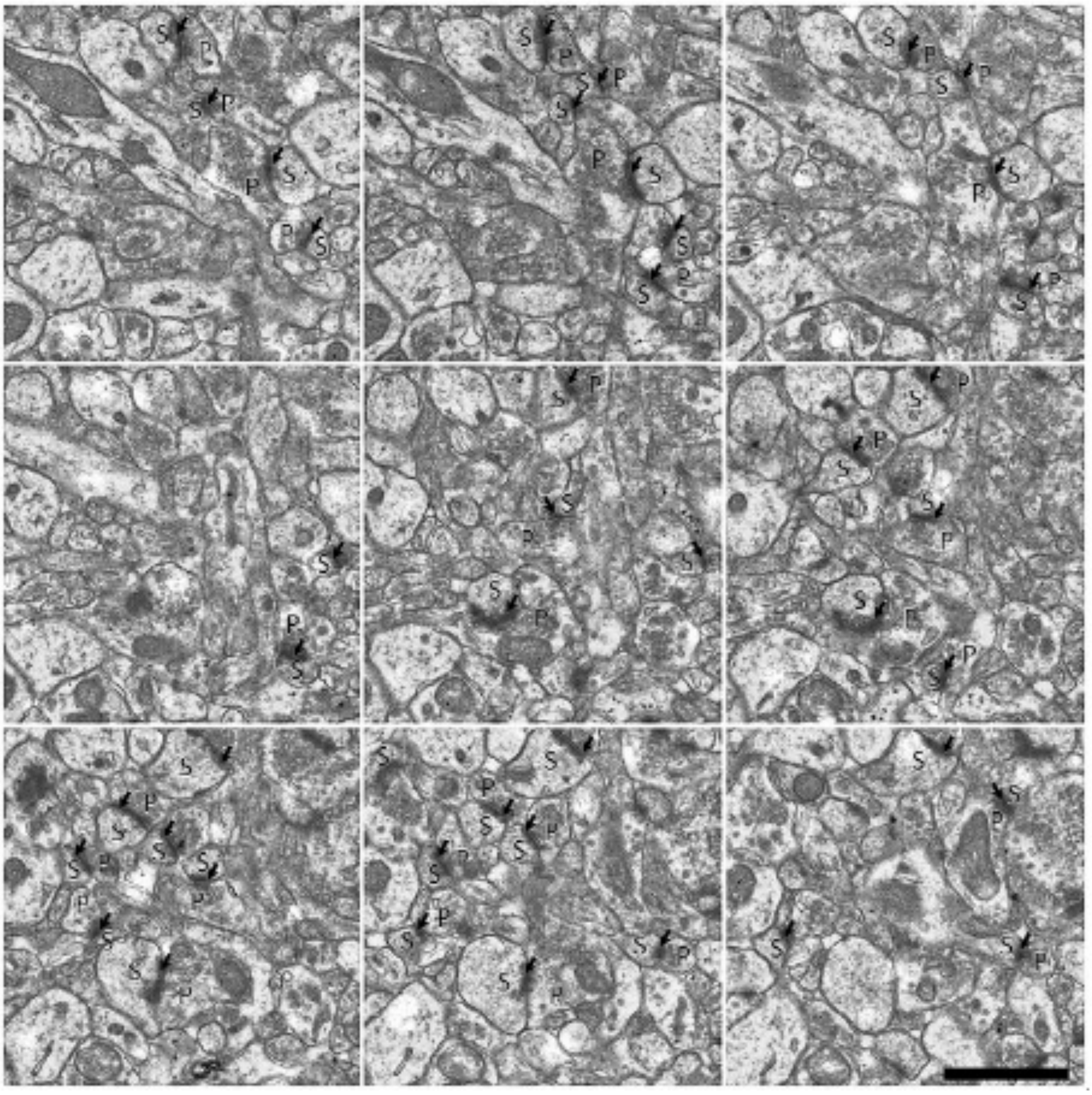

Figure S1. Representative serial electron micrographs, showing excitatory synapses. Related to Figure 2. Scale bar =1 µm. Arrows indicate postsynaptic densities (PSD), spines (S) and presynaptic boutons (P).

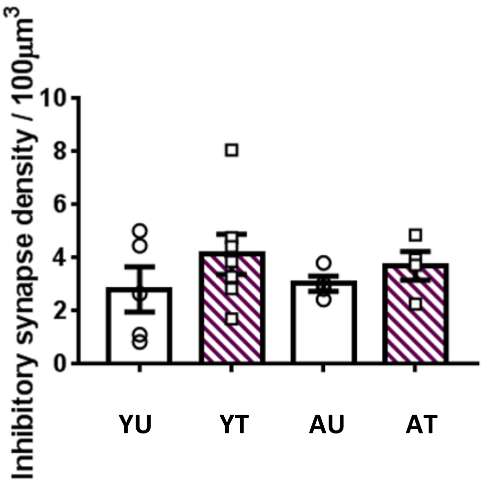

Figure S2. No change in inhibitory synapse density after CFC in young and aged mice. Related to Figure 2. Inhibitory synapse density in hippocampal CA1 stratum radiatum (SR) was not significantly changed 24 after CFC (Figure 1A) in both young and aged mice (n_{YU}=5, n_{YT}=7, n_{AU}=4, n_{AT}=4, effect of age $F_{(1,16)}=0.02$, p=0.88; effect of CFC $F_{(1,16)}=1.74$, p=0.20; interaction CFC x age $F_{(1,16)}=0.18$, p=0.67). Mean ± standard error of mean. Individual data plots representing each animal within group overlay the bar graphs.

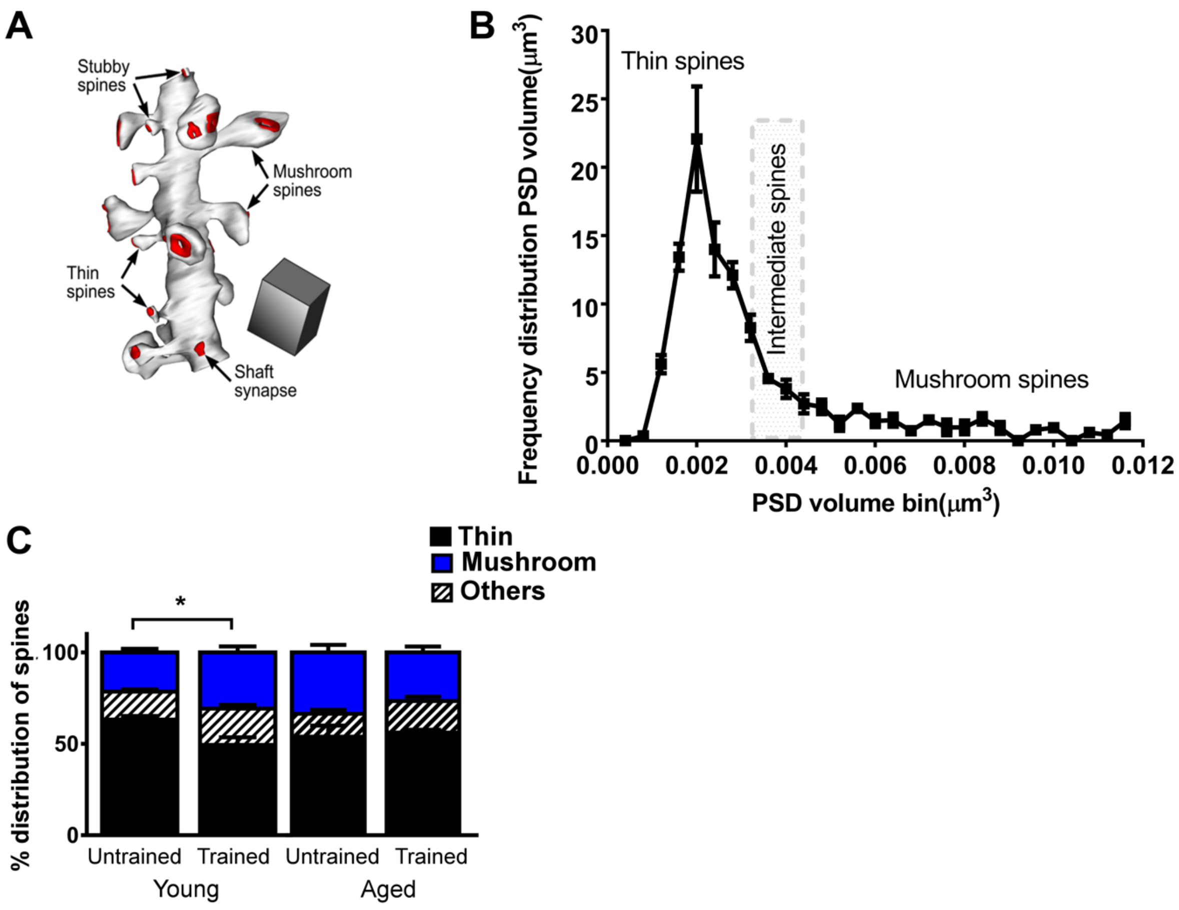

Figure S3. In young, but not aged mice contextual fear memory formation is associated with increase in mushroom spines. Related to Figure 2. (A) An example for a 3D reconstruction, which was used to assess morphology and density of dendritic spines in hippocampal CA1 SR before and 24 h after CFC. Scale bar =1 μm^3. **(B)** Frequency distribution of dendritic spine morphology for young untrained mice is shown. For frequency distribution analysis dendritic spines were categorized according to postsynaptic density volume (see, STAR Methods). Briefly, we have examined the three classes of spine synapses: thin spines with PSD volume <0.0032 μm^3, intermediate spines, which are spines of big size but without complex PSD and spine apparatus (which are the main properties of mushroom spines) with PSD volume 0.0032–0.0044 μm^3; and mushroom spines PSD volume >0.0044 μm^3. **(C)** In young, but not aged mice contextual fear memory formation was associated with an increase in mushroom-type spines at the expense of thin spines (n_{YU}=4, n_{YT}=5, n_{AU}=4,

n_{AT}=4; ratio thin spines to mushroom spines: effect of age $F_{(1,13)}$=1.03, p=0.33; effect of CFC $F_{(1,13)}$=1.06, p=0.32; interaction CFC x age $F_{(1,13)}$= 4.80, p<0.05; Tukey's post hoc tests: YU vs YT p<0.05; AU vs AT, p=0.44).

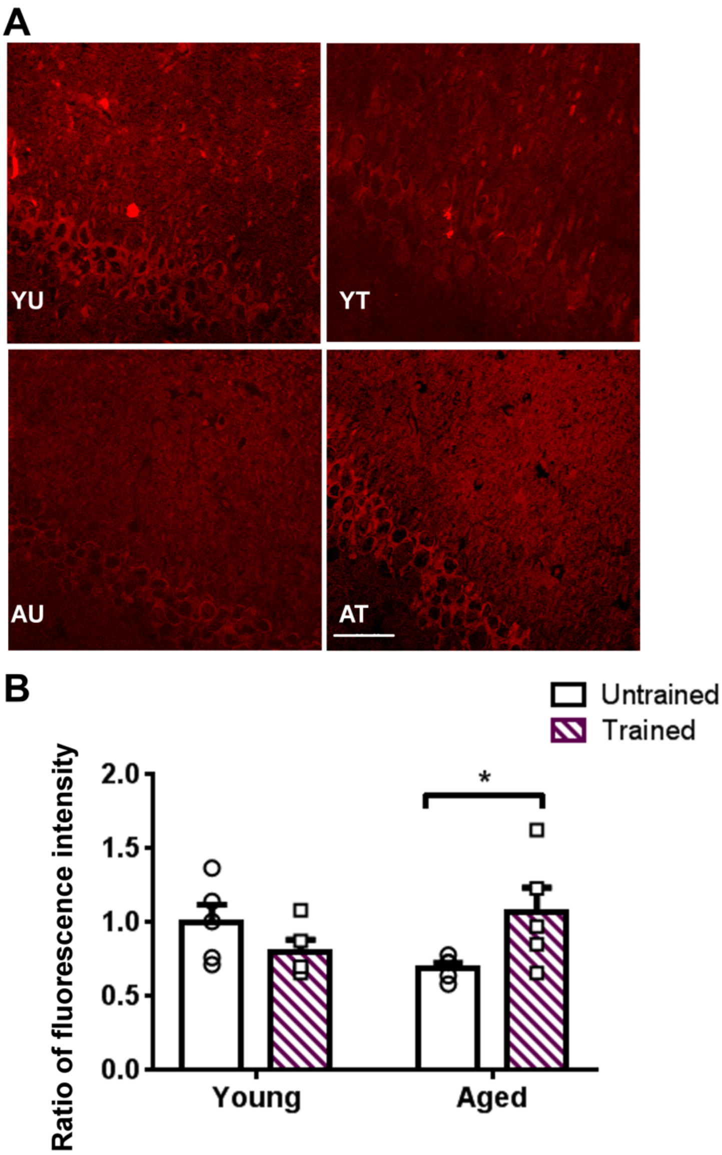

Figure S4. In aged, but not young mice contextual fear memory formation is associated with an increase of total αCaMKII levels. Related to Figure 2. (**A**) Representative immunohistochemical images of total αCaMKII in hippocampal CA1 SR, stratum pyramidale (SP) and stratum oriens (SO) in young and aged mice before or 2 h after CFC (Figure 1A). The fluorescence intensity in SR was referred to SO and was normalized to young untrained mice (see, STAR Methods). Scale bar=20 µm. (**B**) CFC increased total αCaMKII levels in aged, but not young mice, suggesting a compensation for reduced autophosphorylation of αCaMKII (Figure 2E) in aged mice (n=5 each, effect of age $F_{(1,16)}=0.034$ p=0.85; effect of

CFC $F_{(1,16)}=0.62$ $p=0.44$; interaction CFC x age, $F_{(1,16)}=6.5$, $p<0.05$; Tukey's post hoc tests: YU vs YT $p=0.23$; AU vs AT $p<0.05$). Mean ± standard error of mean, * ($p<0.05$). Individual data plots representing each animal within group overlay the bar graphs.

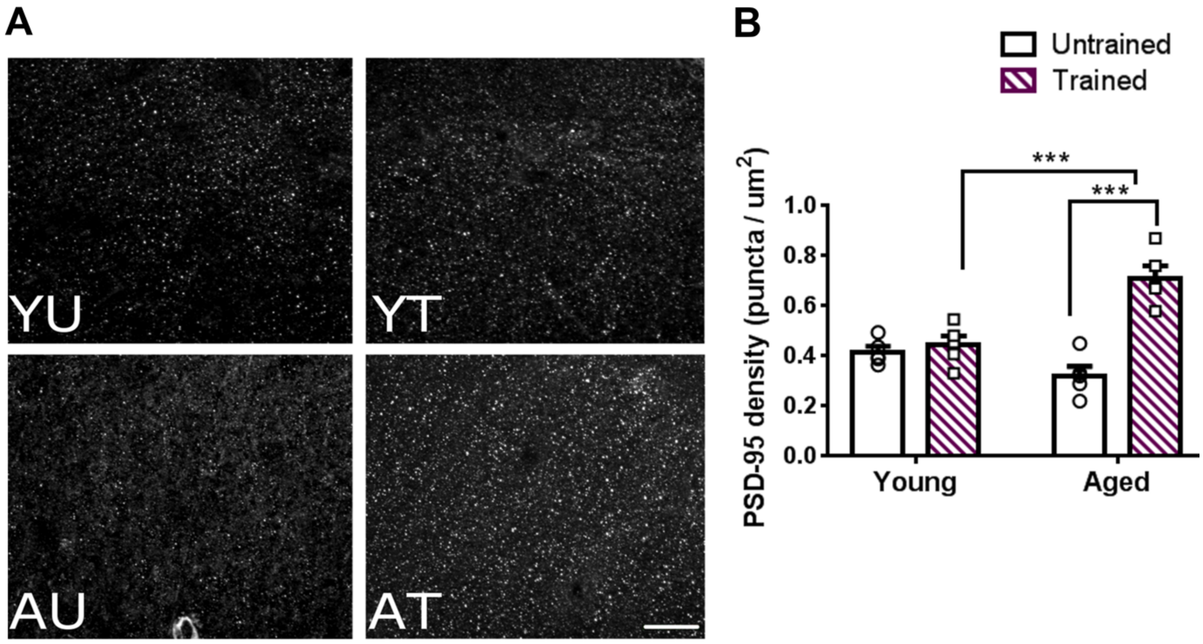

Figure S5. PSD-95 is upregulated after CFC only in aged mice. Related to Figure 4. (A) Representative images of PSD-95 puncta in dorsal hippocampal CA1 SR in young and aged mice before and 2 h after CFC in an independent experiment with single labelling for PSD-95. Scale bar = 20 µm. **(B)** Contextual fear memory formation in aged, but not young mice was associated with PSD-95 up-regulation (n=5 each, effect of age $F(1,16)=5.43$ $p=0.03$; effect of CFC $F(1,16)=30.97$ $p<0.001$; interaction CFC x age $F(1,16)=23.2$ $p<0.001$; Tukey's post hoc tests: YU vs YT $p=0.61$; AU vs AT $p<0.001$; YT vs AT $p<0.001$). Mean ± standard error of mean, *** ($p<0.001$). Individual data plots representing each animal within group overlay the bar graphs.

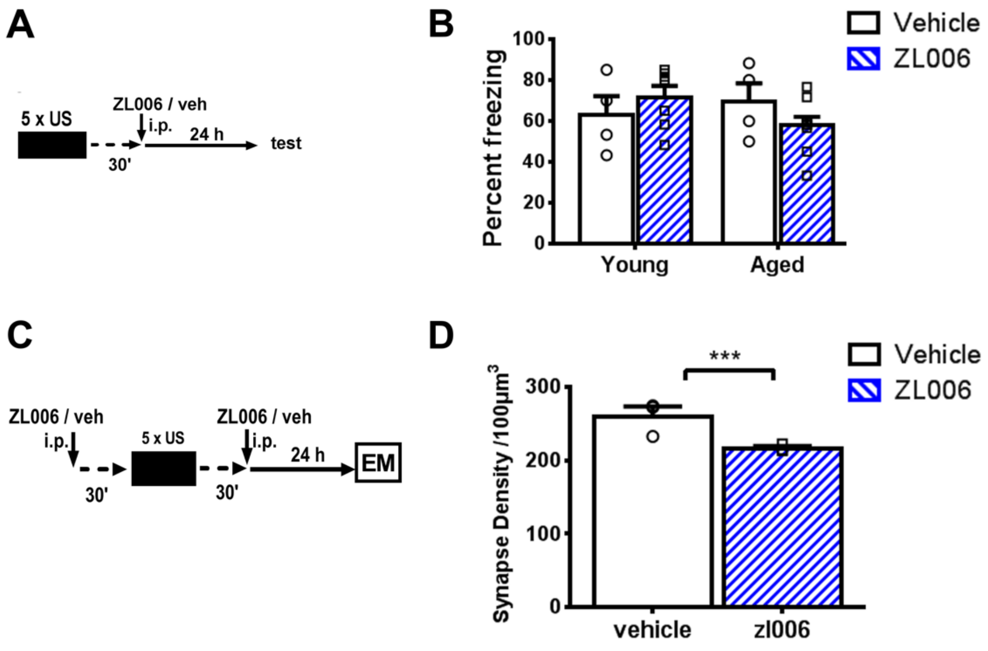

Figure S6. Administration of ZL006 30 minutes after conditioning does not impair contextual fear memory formation in aged mice (A, B), and impact of pre- and post-training ZL006 treatment on the density of excitatory synapses in aged, trained mice (C, D). Related to Figures 5 and 6. **(A)** ZL006 (10 mg/kg, i.p.) or vehicle (0.9% saline and DMSO) was administered to young and aged mice 30 min after CFC and contextual memory was assessed 24 h after CFC. **(B)** This ZL006 treatment did not affect contextual fear memory formation in young and aged mice (n_{Yveh}=4, n_{YZL006}=7, n_{Aveh}=4, n_{AZL006}=9; effect of age $F_{(1,20)}$=0.055, p=0.81; effect of treatment $F_{(1,20)}$=0.30, p=0.58; interaction age x treatment $F_{(1,20)}$=2.48 p=0.13). **(C)** ZL006 (10 mg/kg, i.p.) was administered 30 min before and 30 min after CFC, and 24 h after CFC an EM analysis was carried out. **(D)** ZL006 reduced by about 16% the density of excitatory synapses, including MIS, in area CA1 SR in aged, trained mice (n_{veh}=3, n_{zl006}=3, t(4)=4.22, p<0.001). Mean ± standard error of mean. *** (p<0.001). Individual data plots representing each animal within group overlay the bar graphs.

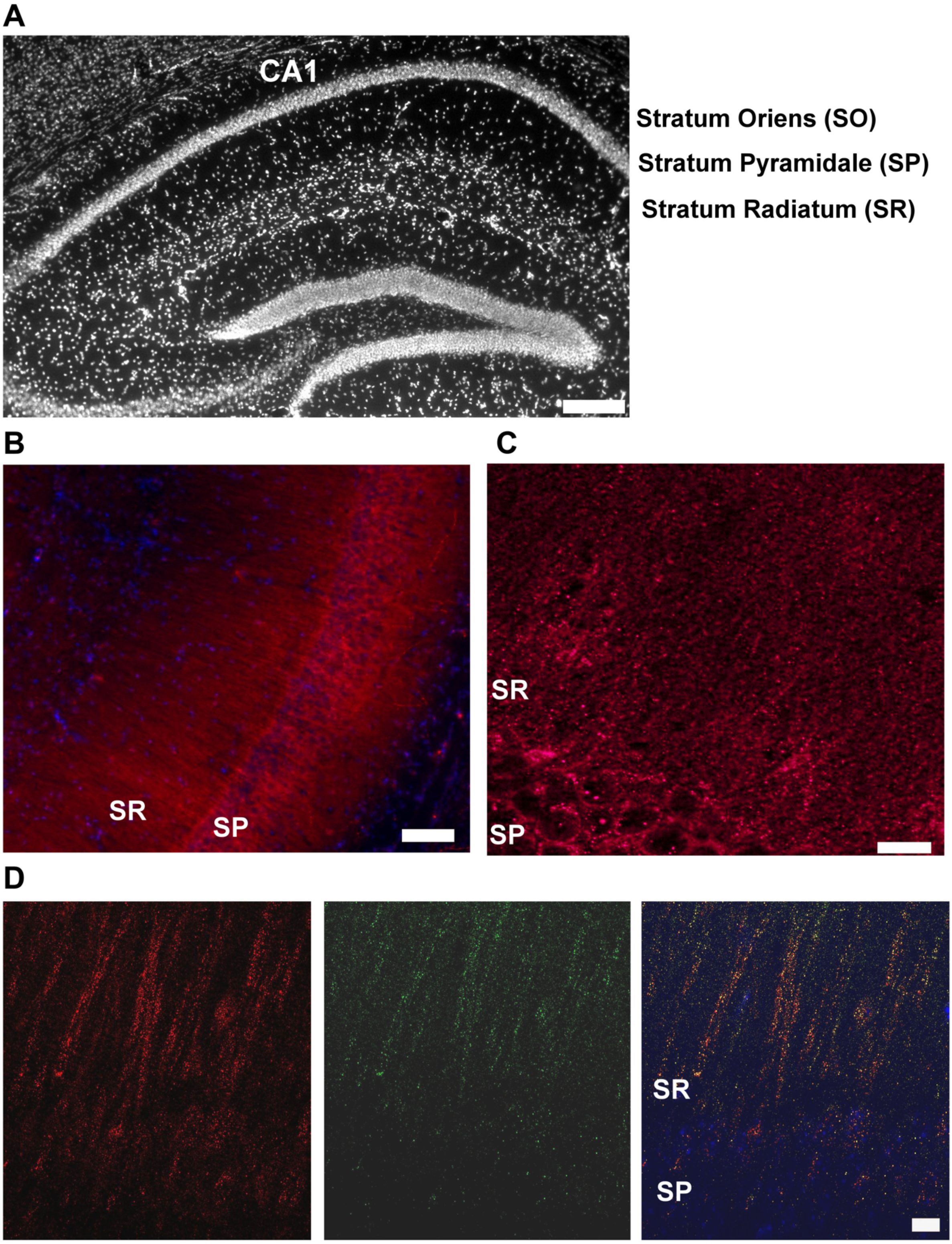
A
CA1
Stratum Oriens (SO)
Stratum Pyramidale (SP)
Stratum Radiatum (SR)
B
SR
SP
C
SR
SP
D
SR
SP

Figure S7. Representative immunohistochemistry images showing landmarks. Related to Figures 4 and 6, and STAR Methods (Light microscopy and image analysis). (A) Representative low magnification image of DAPI-stained dorsal hippocampus, showing stratum oriens (SO), stratum pyrmidale (SP), and stratum radiatum (SR) within the CA1 region. **(B, C)** Representative immunostaining of PSD-95 (red) and DAPI counterstaining (blue) at low magnification showing SP and SR. **(B)** depicts that staining was reliably within SR, and higher magnification in **(C)** shows representative PSD-95 puncta. **(D)** Example of sequential labeling for nNOS (red) and PSD-95 (green), and counterstaining with DAPI (blue). SP is shown as landmark reference. PSD-95 and nNOS labeling appear as puncta in SR in a clear dendritic pattern. The images were taken at 63x magnification to resolve the puncta. For every image analyzed landmarks, such as SP, were confirmed with DAPI staining at low magnification. Scale bars = 200, 100, 50 and 10 μm, respectively.

Group	Mushroom spines number ± SEM
Young untrained	396,395,020 ± 36,290,215
Young trained 24 h	569,062,129 ± 60,729,639
Aged untrained	560,572,645 ± 67,893,895
Aged trained 24h	468,096,999 ± 55,508,083

Table S1. Estimates of total number of mushroom spines in CA1 stratum radiatum before and after CFC in young and aged mice. Total number of mushroom spines in CA1 stratum radiatum was estimated from mushroom spines and measured CA1 stratum radiatum volume. The estimation suggests that in young mice CFC may result in generation of tenths of millions of mushroom spines, which does not occur in aged animals.

Group	MIS number ± SEM
Young untrained	1,756,714 ± 616,963
Young trained 24 h	3,798,349 ± 544,215
Aged untrained	4,897,579 ± 907,087
Aged trained 24h	9,736,155 ± 942,931

Table S2. Estimates of total number of multi-innervated spines (MIS) in CA1 stratum radiatum before and after CFC in young and aged mice. Total number of MIS in CA1 stratum radiatum was estimated from MIS density and measured CA1 stratum radiatum volume. In aged mice CFC may result in generation of several million MIS.
